# Metabolic and Inflammatory Adverse Drug Reactions Associated with Amlodipine: A Descriptive and Disproportionality Analysis of EudraVigilance Reports

**DOI:** 10.3390/ph19081177

**Published:** 2026-07-27

**Authors:** Crina Cristina Solomon, Anca Butuca, Adina Frum, Carmen Maximiliana Dobrea, Claudiu Morgovan, Nastaca Alina Palade, Alina Liliana Pintea, Dragoș Anton Dădârlat, Steliana Ghibu, Florina Batar, Mariana Cornelia Tilinca, Felicia Gabriela Gligor

**Affiliations:** 1 Preclinical Department, Faculty of Medicine, “Lucian Blaga” University of Sibiu, 550169 Sibiu, Romania; crinacristina.solomon@ulbsibiu.ro (C.C.S.); carmen.dobrea@ulbsibiu.ro (C.M.D.); claudiu.morgovan@ulbsibiu.ro (C.M.); nastacaalina.coman@ulbsibiu.ro (N.A.P.); florina.batar@ulbsibiu.ro (F.B.); felicia.gligor@ulbsibiu.ro (F.G.G.); 2 County Emergency Clinical Hospital Sibiu, 550025 Sibiu, Romania; alina-liliana.pintea@ulbsibiu.ro; 3 Department of Dental Medicine and Nursing, Faculty of Medicine, “Lucian Blaga” University of Sibiu, 550169 Sibiu, Romania; dragos.dadarlat@ulbsibiu.ro; 4 Department of Pharmacology, Physiology and Pathophysiology, Faculty of Pharmacy, “Iuliu Haţieganu” University of Medicine and Pharmacy, 6A Louis Pasteur Street, 400349 Cluj-Napoca, Romania; stelianaghibu@yahoo.com; 5 Department of Internal Medicine I, Faculty of Medicine in English, “George Emil Palade” University of Medicine, Pharmacy, Science and Technology, 540142 Targu Mures, Romania; mariana.tilinca@umfst.ro; 6 Clinical Compartment of Diabetes Mellitus, Nutrition and Metabolic Diseases, Emergency Clinical County Hospital of Targu Mures, 540136 Targu Mures, Romania

**Keywords:** amlodipine, pharmacovigilance, EudraVigilance, metabolic syndrome, dysglycemia, inflammation

## Abstract

**Background/Objectives**: The global rise in obesity-related hypertension, metabolic syndrome, and chronic inflammation calls for a precise characterization of the safety profiles of first-line therapies. While amlodipine is considered metabolically neutral, its real-world impact on dysglycemia and inflammatory biomarkers remains incompletely defined. This study aims to characterize the metabolic and inflammatory adverse drug reaction profile of amlodipine, using the EudraVigilance database. **Methods**: Descriptive and disproportionality analyses were performed on 41,872 Individual Case Safety Reports recorded prior to 17 May 2026. Amlodipine was compared against major antihypertensive classes (beta-blockers, angiotensin-converting enzyme (ACE) inhibitors, sartans, and diuretics), calculating reporting odds ratios (ROR) and 95% confidence intervals. **Results**: “Hyperglycaemia” could be considered a safety signal for amlodipine, compared to beta-blockers (e.g., bisoprolol—ROR: 2.56), ACE inhibitors (e.g., ramipril—ROR: 2.82), and sartans (e.g., candesartan—ROR: 3.85). “Metabolic syndrome” was reported for amlodipine with a lower probability than for hydrochlorothiazide (ROR: 0.35). Inflammatory signals (e.g., increased C-reactive protein) appeared less frequently for amlodipine than for certain renin–angiotensin–aldosterone system inhibitors (e.g., perindopril—ROR: 0.53). **Conclusions**: The disproportionality analysis identified relatively lower reporting frequencies for several metabolic and inflammatory adverse drug reactions, compared with selected antihypertensive agents. These findings represent pharmacovigilance signals that warrant further investigation in analytical epidemiological and clinical studies. Although hyperglycemia was reported disproportionally relative to several comparator drugs, reports of T2DM were less frequently reported for amlodipine. However, these observations should not be interpreted as evidence of differences in clinical risk, because disproportionality analyses cannot establish incidence or causality. Reports of inflammation likely reflect patient comorbidities rather than a direct drug effect. These findings demonstrate that post-marketing surveillance remains essential, even when accounting for the methodological limitations of spontaneous reporting.

## 1. Introduction

Arterial hypertension coupled with type 2 diabetes mellitus (T2DM) is composed of the two leading modifiable risk factors for the incidence of cardiovascular disease (CVD) globally [1]. Also, the increasing prevalence of obesity and obesity-related hypertension parallels the growing epidemic of metabolic syndrome (MS) and type 2 diabetes mellitus (T2DM) [2]. In addition, the pathophysiological relationships among metabolic risk factors such as obesity and diabetes, chronic kidney disease and the cardiovascular system have led to the conceptualization of the novel cardiovascular–kidney–metabolic syndrome by the AHA, as well as growing appreciation of the confluence of these factors and its profound impacts on morbidity and mortality outcomes [3]. In these context, excess and dysfunctional adipose tissue (particularly visceral adiposity and other ectopic fat deposition) can cause inflammation, insulin resistance and the emergence of metabolic risk factors and countless systemic effects, including an increased risk for CVD [4].

The rise in the prevalence of these cardiometabolic diseases has led to an increase in the number of people living with concurrent hypertension and T2DM [5]. Approximately 60–80% of individuals with T2DM have concomitant hypertension, while hypertension and MS frequently coexist and together represent a major risk factor for CVD morbidity and mortality worldwide [6].

Given the rising burden of hypertension and diabetes mellitus, there is currently a great need to understand the metabolic profiles of antihypertensive drugs that can effectively prevent the onset of T2DM in distinct patient populations. Consequently, current hypertension guidelines emphasize not only effective blood pressure control but also the selection of antihypertensive therapies with favorable metabolic profiles [7]. Although effective blood pressure decrease remains the primary goal, antihypertensive drug classes differ in their effects on glucose metabolism, lipid homeostasis, body weight, and insulin sensitivity, all of which may influence long-term clinical outcomes [8]. While some drug classes, such as thiazide diuretics and conventional beta-blockers (BB), have been associated with impaired glucose metabolism and adverse lipid profile changes, calcium channel blockers (CCBs) are generally regarded as metabolically neutral [9]. However, this “metabolic neutrality” label is largely based on classical endpoints such as new-onset diabetes and lipid parameters, whereas real-world evidence on amlodipine’s effects on dysglycemia and inflammatory biomarkers specifically remains limited; testing the strength of this assumption is a central aim of the present study, and as detailed below, our findings partly challenge it. Indeed, evidence from a meta-analysis showed that CCBs therapy was not associated with a significant increased risk of new-onset T2DM [10]. However, compared with other classes of antihypertensive drugs, CCBs were associated with a higher incidence of T2DM relative to angiotensin-converting enzyme inhibitors (ACEIs) or angiotensin II receptor blockers (ARBs), and a lower incidence compared with BB or diuretics [11]. On the other hand, diuretics have been associated with detrimental effects on glucose metabolism [12]. It would be reasonable to assume that the drug-induced increases in glucose levels and T2DM incidence would have increased CVD risk, similarly to traditional risk factors for new-onset T2DM [13].

CCBs are a heterogeneous class of drugs that are often classified in two major categories based on their chemical structure, namely, (a) dihydropyridine calcium channel blockers (DHP CCBs), such as amlodipine, nicardipine, manidipine and lercanidipine; and (b) non-dihydropyridine calcium channel blockers (non-DHP CCBs), which include verapamil and diltiazem. DHP CCBs and non-DHP CCBs bind to different sites of the alpha-1 subunit of L-type calcium channels [14]. Thanks to these pharmacologic properties, DHP CCBs are mainly used for the treatment of hypertension, atrial fibrillation/atrial flutter, vasospastic angina and chronic stable angina [15]. Moreover, non-DHP CCBs are used for the treatment and prophylaxis of paroxysmal supraventricular tachycardia [16].

However, not all subtypes of the CCBs class have the same effect on glucose homeostasis. Among them, amlodipine has become the first-line prescribed, long acting dihydropyridine CCB due to its multiple pleotropic effects, including anti-atherosclerotic properties, renoprotection, and established cardiovascular benefits [17]. Despite being recommended as a first-line therapy for hypertension, its adverse drug reactions (ADRs) related to dysglycemia, inflammatory disorders, and MS should be under surveillance. A study suggested that, in hypertensive type 2 diabetes patients, certain DHP CCB drugs significantly reduce C-reactive protein (CRP) levels in addition to lowering blood pressure, indicating potential anti-inflammatory benefits and supporting its preferential use over other non-DHP CCBs for blood pressure management in this population [18].

Current hypertension guidelines recommend combination therapy for many patients whose blood pressure is not adequately controlled with monotherapy. CCBs, including amlodipine, are commonly combined with ACEIs, ARBs or thiazide-like diuretics because these combinations provide effective blood pressure reduction and improve cardiovascular outcomes [19]. For example, triple therapy with CCBs, ARBs and diuretics is one of the most effective therapeutic strategies in the treatment of hypertension. The development of modified-release oral formulations containing antihypertensive combinations is an important research direction, as these allow optimal control of the release of active substances, reducing the frequency of administration, improving patient compliance and increasing therapeutic efficacy [20]. Consequently, amlodipine is extensively prescribed both as monotherapy and as part of fixed-dose combination therapies, resulting in substantial patient exposure across diverse clinical settings.

Despite the well-established efficacy and overall safety of amlodipine, its metabolic and inflammatory adverse drug reactions profile in routine clinical practice remains incompletely characterized. The EudraVigilance (EV) database contains spontaneous reports of suspected adverse drug reactions submitted mainly across the European Economic Area (EEA) and represents an important resource for post-marketing drug safety evaluation. Descriptive analyses combined with disproportionality methods can identify potential safety signals and compare reporting patterns between drugs used for similar clinical indications.

Therefore, the present study aimed to characterize the ADRs profile of amlodipine reported in the EV database, with particular emphasis on dysglycemia, inflammatory disorders and MS-related adverse reactions. In addition, comparative disproportionality analyses were performed against commonly prescribed antihypertensive drug classes to better define the metabolic safety profile of amlodipine in routine clinical practice.

## 2. Results

### 2.1. Descriptive Analysis

#### 2.1.1. General Characteristics of Individual Case Safety Reports

According to the data presented in Table 1, 41,872 Individual Case Safety Reports (ICSRs) reported for amlodipine were registered in the EudraVigilance (EV) database. The distribution by age category shows that adverse drug reactions (ADRs) were reported frequently in older people (40.70% in 65–85 years and 7.30 in +85 years category). However, in the active-adults category (18–64 years), an important number of ICSRs was reported (*n* = 14,589, 34.80%). Regarding the sex category, a higher frequency was noticed for females (52.90%) relative to males (43.30%). Also, a high frequency of reporting was observed in non-European Economic Area (EEA) countries (53.70%) compared to EEA countries (46.30%). ICSRs filed by healthcare professionals (HPs) represented a substantial proportion of all reports (77.80%).

#### 2.1.2. Distribution of ICSRs by System Organ Classes

A high frequency of reports was noticed in the following System Organ Classes (SOCs): “General disorders and administration site conditions” (*n* = 14,367, 34.31%), “Nervous system disorders” (*n* = 8400, 20.06%), “Gastrointestinal disorders” (*n* = 7382, 17.63%), “Skin and subcutaneous tissue disorders” (*n* = 6524, 15.58%), and “Vascular disorders” (*n* = 6331, 15.12%) (Table 2).

#### 2.1.3. Analysis of Reporting Behavior

HPs registered more than 60% of the total reports in each SOC, except for “Product issues” (*n* = 358, 53.00%), “Eye disorders” (*n* = 1012, 56.90%) and “Ear and labyrinth disorders” (*n* = 457, 48.7%) (Table 3).

#### 2.1.4. Analysis of Serious–Non-Serious Cases

The highest frequency of serious ADRs were reported for amlodipine in “Neoplasms benign, malignant and unspecified (incl cysts and polyps)” (*n* = 860, 98.85%), “Surgical and medical procedures” (*n* = 427, 97.49%), “Endocrine disorders” (*n* = 265, 97.07%), “Hepatobiliary disorders” (*n* = 1251, 96.08%), and “Congenital, familial and genetic disorders” (*n* = 167, 95.98%). Contrastingly, a lower frequency of serious ADRs was noticed for “Product issues” (*n* = 374, *n* = 55.41%), “Ear and labyrinth disorders” (*n* = 554, 59.00%), “General disorders and administration site conditions” (*n* = 9086, 63.24%), “Skin and subcutaneous tissue disorders” (*n* = 4169, 63.90%), and “Reproductive system and breast disorders” (*n* = 456, 65.52%) (Table 4).

#### 2.1.5. Preferred Terms Analysis

In the Dysglycaemia and Type 2 Diabetes category, the highest number of reports were registered for “Hyperglycaemia” (*n* = 153) and “Type 2 diabetes mellitus” (*n* = 54). However, the pre-diabetic state related to amlodipine was noticed in two Preferred Terms (PTs) (Table 5): “Glucose tolerance impaired” (*n* = 15) and “Impaired fasting glucose” (*n* = 8). ADRs suggestive of inflammatory processes were reported under some PTs, always with a high number of cases: “C-reactive protein increased” (*n* = 88) and “Inflammation” (*n* = 77). Regarding PTs referred to metabolic syndrome and obesity-related disorders, “hepatic steatosis” (*n* = 52), “blood triglycerides increased” (*n* = 37) and “obesity” (*n* = 36) were the ones most frequently used.

### 2.2. Disproportionality Analysis

#### 2.2.1. Dysglycaemia and Type 2 Diabetes Mellitus Spectrum

Reporting odds ratio (ROR) and 95% confidence interval (95% CI) were calculated. Regarding the “glucose tolerance impaired” PTs, a different statistical reporting probability was not observed for amlodipine, except in comparison with valsartan (ROR: 0.44; 95%CI: 0.19–1.00) (Table 6 and Supplementary Material—Table S1). According to the results presented in Table 6, “Hyperglycaemia” could be considered a safety signal for amlodipine by comparison with (i) beta-blockers: metoprolol (ROR: 1.64; 95%CI: 1.18–2.29) and bisoprolol (ROR: 2.56; 95%CI: 1.69–3.86); (ii) ACEIs: enalapril (ROR: 2.54; 95%CI: 1.62–3.99), perindopril (ROR: 5.33; 95%CI: 2.49–11.40), lisinopril (ROR: 1.74; 95%CI: 1.21–2.50), and ramipril (ROR: 2.82; 95%CI: 1.93–4.13); and (iii) sartans: candesartan (ROR: 3.85; 95%CI: 2.18–6.80). Contrastingly, “type 2 diabetes mellitus” (T2DM) was reported for amlodipine with a lower probability than for metoprolol (ROR: 0.46; 95%CI: 0.31–0.70), lisinopril (ROR: 0.21; 95%CI: 0.14–0.30), ramipril (ROR: 0.10; 95%CI: 0.07–0.14), candesartan (ROR: 0.05; 95%CI: 0.04–0.07), valsartan (ROR: 0.37; 95%CI: 0.24–0.56), and indapamide (ROR: 0.48; 95%CI: 0.26–0.89) (Table 6).

#### 2.2.2. Inflammatory Disorders and Inflammation Biomarkers

The probability of a report of “C-reactive protein increased” by an HP is higher for amlodipine than for metoprolol (ROR: 2.11; 95%CI: 1.32–3.38), but less for amlodipine than for some of the inhibitors of RAAS: ramipril (ROR: 0.28; 95%CI: 0.22–0.36), candesartan (ROR: 0.11; 95%CI: 0.09–0.14), and valsartan (ROR: 0.28; 95%CI: 0.21–0.37). Similarly, inflammation was reported with a lower probability for RAAS inhibitors, specifically, perindopril (ROR: 0.53; 95%CI: 0.33–0.84), ramipril (ROR: 0.13; 95%CI: 0.09–0.17), candesartan (ROR: 0.06; 95%CI: 0.05–0.09), and valsartan (ROR: 0.43; 95%CI: 0.29–0.64), but with a high probability than for enalapril (ROR: 2.57; 95%CI: 1.22–5.41). For the PT “inflammatory marker increased”, a reportable number of ICSRs (*n* ≥ 5) was available only for bisoprolol and ramipril among the full comparator list; for these two drugs, no statistically significant differences in reporting were noticed relative to amlodipine (Table 7 and Supplementary Material—Table S2).

#### 2.2.3. Metabolic Syndrome and Obesity-Related Disorders

HPs reported “hepatic steatosis” more frequently for amlodipine than ramipril (ROR: 2.00; 95%CI: 1.09–3.68). Amlodipine had a significantly lower probability as to reports of “blood triglycerides increased” than metoprolol (ROR: 0.41; 95%CI: 0.24–0.69) and valsartan (ROR: 0.36; 95%CI: 0.21–0.62). “Metabolic syndrome” was reported for amlodipine with a lower probability than for hydrochlorothiazide (ROR: 0.35; 95%CI: 0.14–0.92). For amlodipine, it was observed that there was a lower probability of association with “obesity”, relative to metoprolol (ROR: 0.58; 95%CI: 0.35–0.96), lisinopril (ROR: 0.28; 95%CI: 0.18–0.44), ramipril (ROR: 0.11; 95%CI: 0.07–0.16), and candesartan (ROR: 0.05; 95%CI: 0.03–0.07). No differences were registered between amlodipine and any of the other drugs of interest for association with “waist circumference increased” (Table 8 and Supplementary Material—Table S3).

## 3. Discussion

This targeted pharmacovigilance study on amlodipine identified 41,872 ICSRs, with the highest prevalence of reports being observed in the older population, namely, 40.70% in individuals aged 65–85 years, 7.30% in those aged over 85 years (Table 1). Approximately one-third of the reports involved adults 18–64 years old, whereas children and adolescents accounted for less than 1% of the reports. This pattern may be explained by prescribing practices, as several international guidelines, such as that of the European Society for Hypertension, recommend CCBs for older patients [1,21], and the National Institute for Health and Care Excellence (NICE) of the United Kingdom mentions them as first-line therapy for patients over 55 years old, whereas in children, CCBs are second- or even third-line therapies [22]. These recommendations are supported by clinical trials [23], demonstrating the significant benefits of amlodipine relative to stroke outcomes in older populations, as well as by the pathology mechanism of hypertension in older patients, which is characterized by arterial stiffness and low renin levels [24]. Furthermore, in the older population, age-related physiological changes, such as low hepatic clearance of amlodipine due to reduced hepatic blood flow and reduced activity of CYP3A4 [25,26,27], result in a prolonged half-life (up to 50% longer), and accumulation occurs, contributing to increased risk of ADRs [27,28]. Because CYP3A4 is an inducible enzyme involved in the metabolism of different drugs, and polypharmacy is common situation among older adults, amlodipine plasma levels above the therapeutic interval can occur when it is coadministered with CYP3A4 inhibitors or competitors’ substrates [28,29].

The frequency of reports related to women (52.9%) was observed to be slightly higher than the one for men (43.3%). These results are in line with the current state of knowledge, sex-related differences having been previously identified [9]. The difference may be explained by the higher plasmatic concentrations achieved in women receiving the standard 5 mg or 10 mg dose of amlodipine, compared to the same treatment in men, who generally have a higher body weight [25,29,30,31]. Peripheral oedema is one of the common ADRs of CCBs. In clinical trials, women were found to demonstrate oedema more frequently than men during amlodipine treatment, a possible explanation being the hormonally driven higher capillary permeability [30,32,33,34].

Regarding geographic distribution, similar values were recorded for EEA (46.30%) and non-EEA (53.70%) countries. These findings were expected, as amlodipine is included in the World Health Organization’s List of Essential Medicines [34]. Clinical practice guidelines for the management of cardiovascular diseases provide clear recommendations for the prescription of amlodipine [21,22,35]. Its benefits and risks have been a topic of interest for scientists around the world [30,34].

Most reports (77.80%) were submitted by healthcare professionals (HPs), such as doctors, nurses, pharmacists, thereby enhancing the readability of the reported information. It is worthy of note that European health authorities encourage spontaneous reporting by both HPs and patients through accessible electronic reporting systems [36], which may explain why approximately one-fifth of all reports were submitted by members of the general public.

The five most frequently reported SOCs, were, in descending order: “General disorders and administration site conditions” (*n* = 14,367, 34.31%), including the common symptoms tiredness and asthenia, and the generally recognized peripheral oedema [37,38]; “Nervous system disorders” (*n* = 8400, 20.06%), mainly represented by headaches and dizziness [37,38]; “Gastrointestinal disorders” (*n* = 7382, 17.63%), which referred mainly to nausea and abdominal pain [37]; “Skin and subcutaneous tissue disorders” (*n* = 6524, 15.58%), which named mostly rash and flushing [37,38]; and “Vascular disorders” (*n* = 6331, 15.12%). The ADRs included in the latter SOCs are directly linked to amlodipine’s mechanism of action, which causes low blood pressure and temporary redness in the teguments [37]. The most frequently reported ADRs within these SOCs are consistent with the ones mentioned in the Summary of the Product Characteristics (SmPC) [37].

An interesting perspective on how patients perceive the ADRs is shown when comparing reports submitted by HPs and non-HPs. Although similar reporting rates were observed for “Eye disorders” and “Ear and labyrinth disorders”, these ADRs are among the most frequently reported in the non-HP category, while they appeared at much lower rates in reports from HPs, who often consider them to be more subjective symptoms. A similar reporting pattern between HP and non-HP reporters regarding tinnitus has also been observed with bisoprolol [39]. Patients also report “Product issues”, such as packaging defects or inconsistencies in appearance. On the other hand, “Psychiatric disorders” were reported by HPs in 4.165 out of 5.275 cases, suggesting that conditions such as severe anxiety, sleep disorders or mood disturbances typically require clinical evaluation before being classified as ADRs [37].

Taking into consideration the documented safety profile of amlodipine (consisting mostly of mild ADRs [40,41]), the serious versus non-serious case frequency should be interpreted based on the premise that HPs have a tendency to report serious cases, to the detriment of non-serious ones [42,43,44,45]. With high frequency, the reported serious cases pointed to Hepatobiliary disorder (*n* = 1251, 96.08%), already recognized by SmPC as an extremely rare ADR [37,38]. Although they do not present often, when diagnosed, these disorders require hospitalization [46,47].

There are several amlodipine ADRs included in the Dysglycaemia and T2DM category, the most reported being “Hyperglycaemia” (*n* = 153). A speculative mechanistic explanation, derived from two individual case reports of supratherapeutic amlodipine ingestion, is that amlodipine’s blockade of L-type calcium channels may, especially in overdoses, impair pancreatic beta-cell insulin secretion and thereby induce hyperglycemia, as described by DeGeeter [48] and Kumar [49]. It should be emphasized that this mechanism was described in the context of acute, overdose exposure, and its extrapolation to the pattern of hyperglycemia reporting observed in routine, population-level spontaneous reporting is speculative and should be interpreted with caution.

Regarding insulin resistance, several studies suggest that amlodipine may improve insulin sensitivity or have a neutral effect on glucose metabolism, although the findings are not entirely consistent across populations and study designs [50,51].

Although, in the Inflammatory Disorders & Biomarkers category, “C-protein increased” was the most frequently reported event (*n* = 88), other studies have suggested that amlodipine may reduce high-sensitivity CRP [52]. This may indicate that the elevated results in our study may be related to measurements being done while either concomitant ADRs such as oedema, rash, and vasculitis were present, or underlying infection, inflammation or comorbidity, rather than the amlodipine itself.

In the Metabolic Syndrome & Obesity-Related Disorders category, “Hepatic steatosis” was the PT of interest reported the most (*n* = 41). However, this finding should be interpreted cautiously because spontaneous reporting systems are designed to detect safety signals rather than establish causal relationships. Hepatic steatosis is highly prevalent among patients with hypertension, obesity, T2DM, dyslipidemia, and metabolic syndrome, conditions that frequently coexist in individuals receiving amlodipine. Moreover, the current clinical literature does not identify hepatic steatosis as a recognized adverse reaction to amlodipine [46]. Reported cases of amlodipine-induced liver injury are rare and are predominantly characterized by hepatocellular, cholestatic, or mixed patterns of injury rather than steatosis [53]. Furthermore, amlodipine was reported to improve parameters of fatty liver in both animal models [54,55] and humans [56]. Applying the pre-specified EMA criteria, the amlodipine versus ramipril comparison for hepatic steatosis (ROR: 2.00; 95% CI: 1.09–3.68; *n* = 52) technically constitutes a disproportionality signal. Nevertheless, we discuss this finding with more caution than the hyperglycemia signal and do not treat it as being equally supportive of causality. This differentiation is justified by external evidence: while a substantial body of preclinical and clinical data contradicts amlodipine’s association with hepatic steatosis, no such contradictory data exist for hyperglycemia. This nuanced interpretation reflects the weight of available external evidence for each PT, rather than an inconsistent application of our filtering criteria.

The dataset shows there is a higher probability of reporting hyperglycemia for amlodipine in direct comparisons with the main beta-blockers and inhibitors of the renin–angiotensin–aldosterone system (ACE inhibitors and candesartan). This observation could be explained by amlodipine’s capacity to block calcium L channels, an effect that, mostly in amlodipine overdoses, extends to the pancreas, blocking insulin secretion, which is clinically expressed as “Hyperglycaemia” [48].

The probability of reporting “Type 2 diabetes” as an ADR was lower for amlodipine than for the specific comparators: metoprolol, lisinopril, ramipril, candesartan, and valsartan.

The analysis of the two result sets would suggest amlodipine has the possibility to cause a transient increase in hyperglycemia, a sign of great significance for the HP, one which is easily quantifiable through routine blood tests and triggers report submission. However, when analyzing the progression to a formal diagnosis of T2DM, amlodipine ranks statistically much better than most comparators, a situation in line with the clinical study ASCOT-BPLA, where therapeutic regimens based on amlodipine have been shown to associate a significantly lower risk of developing de novo diabetes compared to those based on beta-blockers [57]. The positioning of beta-blockers and sartans on the higher reporting-probability side compared to amlodipine should also take into consideration confounding by indication situations due to the prescription patterns, as these antihypertensives are preferentially prescribed for patients who have already been diagnosed with metabolic syndrome, obesity or renal impairment, given the documented benefits [58,59]. For consistency, the same reasoning should be applied when the signal points at amlodipine itself: as a low-cost, first-line generic drug, amlodipine is also likely prescribed for a broader and, on average, healthier population, which could similarly inflate its comparatively favorable reporting profile for several PTs. Consequently, the higher probability of reporting hyperglycemia for amlodipine relative to beta-blockers and RAAS inhibitors cannot be fully attributed to a direct drug effect without also considering that confounding by indication may work in the opposite direction here, favoring the comparators rather than amlodipine. This possibility is acknowledged as a limitation in the comparative interpretation.

With regard to pre-diabetes (“Glucose tolerance impaired”), the statistical equivalence with most classes suggests a stable long-term profile. The exception represented by valsartan (where a stronger disproportionality was recorded to the detriment of amlodipine) can be correlated with data from the NAVIGATOR study, which showed that valsartan reduces the incidence of impaired glucose tolerance in patients at high cardiovascular risk [60]. The HP reports in our study also reflect this superior ability of valsartan to manage pre-diabetic conditions, compared to amlodipine.

The results obtained in the Inflammatory Disorders & Biomarkers category may reflect how the prescribing patterns directly influence pharmacovigilance data. The most obvious statistical contrast is shown in the comparison between amlodipine and RAAS inhibitors (ACE inhibitors and sartans). On one hand, amlodipine has a significantly lower probability of reporting “Inflammation” and “C-reactive protein increased” than ramipril, candesartan or valsartan. On the other hand, it shows high disproportionality compared to enalapril. From a pathophysiological point of view, RAAS inhibitors are recognized in the literature for their intrinsic anti-inflammatory properties, capable of lowering serum levels of CRP, IL-6 and TNF-alpha by blocking the angiotensin II axis, a known promoter of vascular oxidative stress [61]. In this clinical context, the “protective” capacity of amlodipine in comparison with ramipril or candesartan is most likely a confounding by indication bias [62,63].

The exception represented by enalapril may be explained by differences in kinetic profile and therapeutic compliance, as enalapril often requires twice-daily administration and has lower plasma stability than new-generation molecules (such as ramipril), which can leave windows of hemodynamic and endothelial imbalance that can mimic or induce acute inflammatory episodes reported as such [64].

The analysis confirms the metabolic neutrality of amlodipine compared to thiazide diuretics (hydrochlorothiazide) and traditional beta-blockers (metoprolol). The observation of a markedly lower probability of reporting “Metabolic syndrome” compared to hydrochlorothiazide is in full agreement with large clinical trials (such as ALLHAT or ASCOT-BPLA) [65]. Thiazide diuretics are known to induce insulin resistance, worsen dyslipidemia and increase the risk of developing metabolic syndrome [66,67,68].

It should be emphasized that the RCT evidence discussed above (ASCOT-BPLA, NAVIGATOR, and ALLHAT) and the present disproportionality findings address fundamentally different questions and cannot be directly compared regarding the nature of the proof they provide: RCTs estimate the incidence of clinical outcomes under controlled trial conditions, whereas disproportionality analysis of spontaneous reports estimates the relative probability of an adverse event being reported under real-world conditions, a quantity influenced by reporting behavior, notoriety, and prescribing patterns rather than incidence alone. Therefore, the RCT findings should be regarded as external evidence that aligns with, and lends biological plausibility to, the pharmacovigilance signals identified here, rather than as evidence that independently confirms or proves them.

Similarly, the lower reporting risk of “Blood triglycerides increased” observed with amlodipine compared with metoprolol may reflect the different metabolic effects of these antihypertensive agents. Conventional β-blockers have been associated with increases in plasma triglyceride levels, an effect attributed to impaired triglyceride clearance resulting from reduced lipoprotein lipase activity and β-adrenergic blockade. In contrast, vasodilating β-blockers exhibit a more favorable metabolic profile [69]. In contrast, amlodipine is generally considered metabolically neutral and has not been shown to adversely affect lipid metabolism. Consequently, the lower reporting frequency of “Blood triglycerides increased” associated with amlodipine in our study is biologically plausible, although causal inferences cannot be drawn from spontaneous reporting data.

Regarding obesity, as clinical guidelines recommend RAAS inhibitors, due to their nephro- and cardioprotective properties [21,22,35], as a first line of treatment in obese patients or patients with advanced metabolic syndrome, these patients—already clinically classified as PT “obesity” by the attending physicians—are much more present in cohorts treated with ramipril or candesartan, causing the appearance of this disproportionate result.

### Limitations of the Study

Once a drug is placed on the market, post-marketing surveillance becomes crucial for continuous monitoring of its safety and efficacy under real-world conditions. This makes it possible to identify rare or long-term side effects that may not have been observed during clinical trials, thus helping to protect patients’ health, and update information on the use of the medicine. Our study used data collected from a large European spontaneous reporting database, EV, which allowed a comparative evaluation of different therapies, providing information leading to a better characterization of their safety profiles under the conditions of current clinical practice. However, there are some limitations that must be recognized in this context. The phenomenon of underreporting and the existence of incomplete reports and duplicates are well-known limitations of pharmacovigilance systems, but other methodological constraints must also be considered. These include the impossibility of establishing a definite causal relationship between the medicinal product and the reported adverse event. Another limitation is that the analysis was based on aggregated data from the publicly accessible EudraVigilance database, which do not provide case-level information on the role of the medicinal product within individual ICSRs. Consequently, the analysis could not be restricted to reports in which the study drugs were classified as suspected medicines, which may have influenced the reported disproportionality estimates. On the other hand, the frequency of reports may be influenced by the notoriety of certain adverse reactions or by the increased interest in some medicines, which may lead to differences in reporting patterns. Also, possible information biases related to patients’ medical history, misclassification, comorbidities, concomitant treatments, or duration of exposure to the drug or the dosage administered are factors that can influence the accuracy and interpretation of pharmacovigilance data. Because all reports available in EudraVigilance were included for each medicinal product, the durations of the reporting period differed according to the marketing history of th comparator drugs. This may have influenced the disproportionality estimates and should be considered when interpreting the results. Another important limitation relates to differences in drug utilization, market share, duration of marketing, and prescribing patterns across antihypertensive classes. Drugs are not prescribed uniformly in clinical practice but are often preferentially selected for specific patient populations according to clinical guidelines and comorbidities. For example, ACEIs and ARBs are frequently prescribed for patients with diabetes, chronic kidney disease, or metabolic syndrome because of their established cardio–kidney protective benefits. Thus, confounding by indication bias may influence reporting patterns and disproportionality estimates independently of the intrinsic safety profile of the drugs. Hence, differences in disproportionality should be interpreted as pharmacovigilance signals rather than direct evidence of comparative safety. However, these limitations highlight the need to supplement pharmacovigilance data with additional information. In this sense, aligning the results with other types of scientific evidence is essential for a more robust and complete understanding of the safety profile.

## 4. Materials and Methods

### 4.1. Study Design

Aggregated data retrieved on the EV database for the period prior to 17 May 2026 (www.adrreports.eu (accessed on 24 May 2026)) were used to perform the descriptive and disproportionality analyses. ICSRs are used to report ADRs in EV [70] and each ICSR includes demographic information (patient’s age and sex), origin of reports (EEA or Non-EEA) and the reporter category (Health Professionals—HP, or Non-HP) [71]. Aggregated data were extracted on 24 May 2026.

To report ADRs, the Medical Dictionary for Regulatory Activities (MedDRA) has established a hierarchical structure with different levels of classification. Thus, a lot of the PTs included in MedDRA could be used to report an ADR. These are subordinated to 27 SOCs, which represent the highest level of hierarchy and include PTs grouped according to the affected system [72].

### 4.2. Descriptive Analysis

General characteristics of ICSRs registered in the EV portal for amlodipine and the distributions of reports by SOC category and the frequency of reports were analyzed [73]. Subsequently, stratified analyses of distribution ADRs by SOC was performed considering the reporting behavior based on the distribution of reports between HPs and non-HPs and, respectively, based on the distribution by seriousness (serious versus non-serious cases). To evaluate the metabolic disturbances associated with amlodipine use, 14 PTs were grouped in three pathophysiological categories (Table 9). The distribution of ADRs between HP and non-HP reporters was evaluated.

### 4.3. Disproportionality Analysis

To evaluate the reporting probability of ADRs associated with amlodipine, disproportionality analysis was performed using comparator drugs selected from the principal antihypertensive classes recommended in contemporary hypertension guidelines. These classes include (i) CCBs (nifedipine, felodipidine), (ii) BBs (metoprolol, carvedilol, bisoprolol, and nebivolol), (iii) ACEIs (enalapril, perindopril, lisinopril, and ramipril), (iv) BRA (candesartan, telmisartan, and valsartan), and (v) diuretics (hydrochlorothiazide, indapamide) [74]. These agents are widely prescribed for the management of hypertension, often for similar clinical indications, and therefore provide clinically relevant comparators for pharmacovigilance signal detection. Comparator drug data were retrieved from the publicly accessible EudraVigilance database by querying the respective active substance name, following the same search strategy and selection procedure used for amlodipine. For each comparator, all reports available in EV at the time of data extraction were included. To improve the quality of analysis, disproportionality results were performed, considering only the HPs’ reports. The ROR and 95% CI were calculated [75]. According to the European Medicine Agency, a signal could be considered disproportionate if the lower bound of the 95% CI is greater than 1 and the number of ICSRs is greater than or equal to 5 [76,77]. Reporting odds ratios (RORs) and corresponding 95% confidence intervals were calculated using MedCalc software version 23.6.3 (MedCalc Software Ltd., Ostend, Belgium).

### 4.4. Ethics

The descriptive and disproportionality analyses use aggregated data published on the EV portal [78]. None of the data refers to any identifiable person, and no personal information was contained in the ICSRs [79]. Therefore, the present study did not require ethics board approval.

## 5. Conclusions

This pharmacovigilance analysis highlights reporting patterns and disproportionality signals related to metabolic and inflammatory ADRs associated with amlodipine in the EudraVigilance database, compared to other major antihypertensive classes. Differences in reporting frequencies were observed relative to selected antihypertensive drugs; however, these findings should be interpreted as hypothesis-generating rather than evidence of comparative clinical safety or causality. The observed reporting patterns may reflect differences in prescribing practices, patient characteristics, and reporting behavior, in addition to potential pharmacological effects. Therefore, the identified signals should be interpreted cautiously and require confirmation in well-designed pharmacoepidemiologic studies and prospective clinical investigations. Briefly, these findings underline the importance of continuous post-marketing surveillance; however, the results must be interpreted within the context of the limitations of spontaneous reporting systems, which do not allow for the establishment of a definite causal relationship.

## Figures and Tables

**Table 1:** General characteristics of ICSRs registered in EV portal.

	*n*	%
Total	41,872	100.0%
Age		
Not Specified	6527	15.6%
0–1 Month	46	0.10%
2 Months–2 Years	115	0.30%
3–11 Years	158	0.40%
12–17 Years	318	0.80%
18–64 Years	14,589	34.80%
65–85 Years	17,040	40.70%
More than 85 Years	3079	7.30%
Sex		
Female	22,153	52.90%
Male	18,131	43.30%
Not Specified	1588	3.80%
Origin		
EEA	19,379	46.30%
NON-EEA	22,493	53.70%
Not Specified	0	
Reporter category		
Healthcare Professional	32,560	77.80%
Non-Healthcare-Professional	9018	21.50%
Not Specified	294	0.70%

**Table 2:** Distribution of reports by SOC category.

	Reports	
SOC	*n*	%
Blood and lymphatic system disorders	1450	3.46%
Cardiac disorders	4463	10.66%
Congenital, familial and genetic disorders	174	0.42%
Ear and labyrinth disorders	939	2.24%
Endocrine disorders	273	0.65%
Eye disorders	1780	4.25%
Gastrointestinal disorders	7382	17.63%
General disorders and administration site conditions	14,367	34.31%
Hepatobiliary disorders	1302	3.11%
Immune system disorders	1102	2.63%
Infections and infestations	2327	5.56%
Injury, poisoning and procedural complications	6269	14.97%
Investigations	5619	13.42%
Metabolism and nutrition disorders	2693	6.43%
Musculoskeletal and connective tissue disorders	5672	13.55%
Neoplasms benign, malignant and unspecified (incl cysts and polyps)	870	2.08%
Nervous system disorders	8400	20.06%
Pregnancy, puerperium and perinatal conditions	229	0.55%
Product issues	675	1.61%
Psychiatric disorders	5275	12.60%
Renal and urinary disorders	2921	6.98%
Reproductive system and breast disorders	696	1.66%
Respiratory, thoracic and mediastinal disorders	4531	10.82%
Skin and subcutaneous tissue disorders	6524	15.58%
Social circumstances	261	0.62%
Surgical and medical procedures	438	1.05%
Vascular disorders	6331	15.12%
Total	41,872	100.00%

**Table 3:** Distribution of reports in SOCs by reporter category.

SOC	*n*			%		
	HP	Non-HP	NS	HP	Non-HP	NS
Blood and lymphatic system disorders	1318	107	25	90.9%	7.38%	1.72%
Cardiac disorders	3397	999	67	76.1%	22.38%	1.50%
Congenital, familial and genetic disorders	158	12	4	90.8%	6.90%	2.30%
Ear and labyrinth disorders	457	472	10	48.7%	50.27%	1.06%
Endocrine disorders	199	71	3	72.9%	26.01%	1.10%
Eye disorders	1012	756	12	56.9%	42.47%	0.67%
Gastrointestinal disorders	5405	1911	66	73.2%	25.89%	0.89%
General disorders and administration site conditions	10,676	3593	98	74.3%	25.01%	0.68%
Hepatobiliary disorders	1165	106	31	89.5%	8.14%	2.38%
Immune system disorders	846	248	8	76.8%	22.50%	0.73%
Infections and infestations	1710	580	37	73.5%	24.92%	1.59%
Injury, poisoning and procedural complications	5134	1102	33	81.9%	17.58%	0.53%
Investigations	4064	1485	70	72.3%	26.43%	1.25%
Metabolism and nutrition disorders	2164	491	38	80.4%	18.23%	1.41%
Musculoskeletal and connective tissue disorders	3772	1876	24	66.5%	33.07%	0.42%
Neoplasms benign, malignant and unspecified (incl cysts and polyps)	573	289	8	65.9%	33.22%	0.92%
Nervous system disorders	5791	2542	67	68.9%	30.26%	0.80%
Pregnancy, puerperium and perinatal conditions	202	25	2	88.2%	10.92%	0.87%
Product issues	358	317	0	53.0%	46.96%	0.00%
Psychiatric disorders	4165	1087	23	79.0%	20.61%	0.44%
Renal and urinary disorders	2242	641	38	76.8%	21.94%	1.30%
Reproductive system and breast disorders	418	271	7	60.1%	38.94%	1.01%
Respiratory, thoracic and mediastinal disorders	3295	1201	35	72.7%	26.51%	0.77%
Skin and subcutaneous tissue disorders	4938	1539	47	75.7%	23.59%	0.72%
Social circumstances	159	101	1	60.9%	38.70%	0.38%
Surgical and medical procedures	287	139	12	65.5%	31.74%	2.74%
Vascular disorders	5061	1214	56	79.9%	19.18%	0.88%

**Table 4:** Distribution of reports in SOCs by seriousness.

SOC	*n*			%		
	Serious	Non-Serious	NS	Serious	Non-Serious	NS
Blood and lymphatic system disorders	1373	69	8	94.69%	4.76%	0.55%
Cardiac disorders	3939	491	33	88.26%	11.00%	0.74%
Congenital, familial and genetic disorders	167	7	0	95.98%	4.02%	0.00%
Ear and labyrinth disorders	554	380	5	59.00%	40.47%	0.53%
Endocrine disorders	265	8	0	97.07%	2.93%	0.00%
Eye disorders	1274	498	8	71.57%	27.98%	0.45%
Gastrointestinal disorders	4910	2428	44	66.51%	32.89%	0.60%
General disorders and administration site conditions	9086	5218	63	63.24%	36.32%	0.44%
Hepatobiliary disorders	1251	45	6	96.08%	3.46%	0.46%
Immune system disorders	983	117	2	89.20%	10.62%	0.18%
Infections and infestations	2100	214	13	90.24%	9.20%	0.56%
Injury, poisoning and procedural complications	5693	575	1	90.81%	9.17%	0.02%
Investigations	4865	724	30	86.58%	12.88%	0.53%
Metabolism and nutrition disorders	2483	204	6	92.20%	7.58%	0.22%
Musculoskeletal and connective tissue disorders	3872	1780	20	68.27%	31.38%	0.35%
Neoplasms benign, malignant and unspecified (incl cysts and polyps)	860	5	5	98.85%	0.57%	0.57%
Nervous system disorders	6229	2133	38	74.15%	25.39%	0.45%
Pregnancy, puerperium and perinatal conditions	216	13	0	94.32%	5.68%	0.00%
Product issues	374	301	0	55.41%	44.59%	0.00%
Psychiatric disorders	4568	694	13	86.60%	13.16%	0.25%
Renal and urinary disorders	2538	378	5	86.89%	12.94%	0.17%
Reproductive system and breast disorders	456	239	1	65.52%	34.34%	0.14%
Respiratory, thoracic and mediastinal disorders	3624	892	15	79.98%	19.69%	0.33%
Skin and subcutaneous tissue disorders	4169	2301	54	63.90%	35.27%	0.83%
Social circumstances	231	30	0	88.51%	11.49%	0.00%
Surgical and medical procedures	427	11	0	97.49%	2.51%	0.00%
Vascular disorders	5294	1016	21	83.62%	16.05%	0.33%

**Table 5:** Distribution of cases by PTs of interest.

Pathophysiologic Category	SOC	PT	HP	Non-HP	NS	Total
Dysglycaemia and Type 2 Diabetes Spectrum	Metabolism and nutrition disorders	Glucose tolerance impaired	12	3	0	15
	Metabolism and nutrition disorders	Hyperglycaemia	136	17	0	153
	Metabolism and nutrition disorders	Impaired fasting glucose	8	0	0	8
	Metabolism and nutrition disorders	Type 2 diabetes mellitus	41	12	1	54
Inflammatory Disorders & Biomarkers	Investigations	C-reactive protein increased	80	5	3	88
	Investigations	Inflammatory marker increased	13	0	0	13
	General disorders and administration site conditions	Inflammation	50	27	0	77
	General disorders and administration site conditions	Systemic inflammatory response syndrome	4	1	0	5
Metabolic Syndrome & Obesity-Related Disorders	Investigations	Blood triglycerides increased	24	12	1	37
	Investigations	High density lipoprotein decreased	18	1	0	19
	Investigations	Waist circumference increased	5	1	0	6
	Metabolism and nutrition disorders	Metabolic syndrome	9	0	0	9
	Metabolism and nutrition disorders	Obesity	30	6	0	36
	Hepatobiliary disorders	Hepatic steatosis	41	9	2	52

**Table 6:** Disproportionality analysis of ADRs related to the Dysglycaemia and T2DM spectrum.

PT	Comparator Drug	ROR Value
Glucose tolerance impaired	Metoprolol	ROR: 0.69; 95%CI: 0.30–1.61
Glucose tolerance impaired	Carvedilol	ROR: 0.42; 95%CI: 0.16–1.06
Glucose tolerance impaired	Enalapril	ROR: 0.98; 95%CI: 0.35–2.80
Glucose tolerance impaired	Lisinopril	ROR: 0.71; 95%CI: 0.29–1.73
Glucose tolerance impaired	Valsartan	ROR: 0.44; 95%CI: 0.19–1.00
Glucose tolerance impaired	Hydrochlorothiazide	ROR: 0.54; 95%CI: 0.21–1.37
Hyperglycaemia	Nifedipine	ROR: 1.23; 95%CI: 0.82–1.82
Hyperglycaemia	Felodipine	ROR: 1.55; 95%CI: 0.68–3.51
Hyperglycaemia	Metoprolol	ROR: 1.64; 95%CI: 1.18–2.29
Hyperglycaemia	Carvedilol	ROR: 0.72; 95%CI: 0.52–1.01
Hyperglycaemia	Bisoprolol	ROR: 2.56; 95%CI: 1.69–3.86
Hyperglycaemia	Enalapril	ROR: 2.54; 95%CI: 1.62–3.99
Hyperglycaemia	Perindopril	ROR: 5.33; 95%CI: 2.49–11.40
Hyperglycaemia	Lisinopril	ROR: 1.74; 95%CI: 1.21–2.50
Hyperglycaemia	Ramipril	ROR: 2.82; 95%CI: 1.93–4.13
Hyperglycaemia	Candesartan	ROR: 3.85; 95%CI: 2.18–6.80
Hyperglycaemia	Telmisartan	ROR: 1.36; 95%CI: 0.81–2.29
Hyperglycaemia	Valsartan	ROR: 1.41; 95%CI: 0.99–2.02
Hyperglycaemia	Hydrochlorothiazide	ROR: 1.00; 95%CI: 0.71–1.40
Hyperglycaemia	Indapamide	ROR: 1.48; 95%CI: 0.85–2.57
Type 2 diabetes mellitus	Nifedipine	ROR: 2.22; 95%CI: 0.88–5.61
Type 2 diabetes mellitus	Metoprolol	ROR: 0.46; 95%CI: 0.31–0.70
Type 2 diabetes mellitus	Carvedilol	ROR: 0.59; 95%CI: 0.33–1.04
Type 2 diabetes mellitus	Bisoprolol	ROR: 1.09; 95%CI: 0.63–1.88
Type 2 diabetes mellitus	Nebivolol	ROR: 0.98; 95%CI: 0.39–2.48
Type 2 diabetes mellitus	Enalapril	ROR: 2.10; 95%CI: 0.99–4.49
Type 2 diabetes mellitus	Perindopril	ROR: 0.93; 95%CI: 0.49–1.78
Type 2 diabetes mellitus	Lisinopril	ROR: 0.21; 95%CI: 0.14–0.30
Type 2 diabetes mellitus	Ramipril	ROR: 0.10; 95%CI: 0.07–0.14
Type 2 diabetes mellitus	Candesartan	ROR: 0.05; 95%CI: 0.04–0.07
Type 2 diabetes mellitus	Telmisartan	ROR: 0.82; 95%CI: 0.38–1.75
Type 2 diabetes mellitus	Valsartan	ROR: 0.37; 95%CI: 0.24–0.56
Type 2 diabetes mellitus	Hydrochlorothiazide	ROR: 1.29; 95%CI: 0.65–2.58
Type 2 diabetes mellitus	Indapamide	ROR: 0.48; 95%CI: 0.26–0.89

**Table 7:** Disproportionality analysis of ADRs related to inflammatory disorders and inflammation biomarkers.

PT	Comparator Drug	ROR Value
C-reactive protein increased	Nifedipine	ROR: 1.35; 95%CI: 0.79–2.31
C-reactive protein increased	Metoprolol	ROR: 2.11; 95%CI: 1.32–3.38
C-reactive protein increased	Carvedilol	ROR: 0.70; 95%CI: 0.45–1.07
C-reactive protein increased	Bisoprolol	ROR: 0.78; 95%CI: 0.55–1.10
C-reactive protein increased	Enalapril	ROR: 1.31; 95%CI: 0.84–2.06
C-reactive protein increased	Perindopril	ROR: 1.15; 95%CI: 0.70–1.90
C-reactive protein increased	Lisinopril	ROR: 0.99; 95%CI: 0.68–1.46
C-reactive protein increased	Ramipril	ROR: 0.28; 95%CI: 0.22–0.36
C-reactive protein increased	Candesartan	ROR: 0.11; 95%CI: 0.09–0.14
C-reactive protein increased	Telmisartan	ROR: 0.64; 95%CI: 0.39–1.04
C-reactive protein increased	Valsartan	ROR: 0.28; 95%CI: 0.21–0.37
C-reactive protein increased	Hydrochlorothiazide	ROR: 1.20; 95%CI: 0.74–1.94
C-reactive protein increased	Indapamide	ROR: 1.35; 95%CI: 0.68–2.70
Inflammation	Nifedipine	ROR: 1.35; 95%CI: 0.69–2.67
Inflammation	Felodipine	ROR: 0.57; 95%CI: 0.24–1.32
Inflammation	Metoprolol	ROR: 0.69; 95%CI: 0.46–1.04
Inflammation	Carvedilol	ROR: 1.74; 95%CI: 0.79–3.85
Inflammation	Bisoprolol	ROR: 1.05; 95%CI: 0.65–1.72
Inflammation	Nebivolol	ROR: 1.19; 95%CI: 0.48–3.00
Inflammation	Enalapril	ROR: 2.57; 95%CI: 1.22–5.41
Inflammation	Perindopril	ROR: 0.53; 95%CI: 0.33–0.84
Inflammation	Lisinopril	ROR: 0.72; 95%CI: 0.46–1.11
Inflammation	Ramipril	ROR: 0.13; 95%CI: 0.09–0.17
Inflammation	Candesartan	ROR: 0.06; 95%CI: 0.05–0.09
Inflammation	Valsartan	ROR: 0.43; 95%CI: 0.29–0.64
Inflammation	Hydrochlorothiazide	ROR: 0.98; 95%CI: 0.56–1.73
Inflammatory marker increased	Bisoprolol	ROR: 0.60; 95%CI: 0.27–1.34
Inflammatory marker increased	Ramipril	ROR: 1.78; 95%CI: 0.63–4.99

**Table 8:** Disproportionality analysis of ADRs related to metabolic syndrome and obesity-related disorders.

PT	Comparator Drug	ROR Value
Hepatic steatosis	Nifedipine	ROR: 2.22; 95%CI: 0.88–5.61
Hepatic steatosis	Metoprolol	ROR: 0.99; 95%CI: 0.60–1.64
Hepatic steatosis	Carvedilol	ROR: 1.25; 95%CI: 0.59–2.67
Hepatic steatosis	Bisoprolol	ROR: 1.73; 95%CI: 0.91–3.29
Hepatic steatosis	Enalapril	ROR: 1.29; 95%CI: 0.69–2.42
Hepatic steatosis	Perindopril	ROR: 1.40; 95%CI: 0.66–2.99
Hepatic steatosis	Lisinopril	ROR: 0.84; 95%CI: 0.51–1.40
Hepatic steatosis	Ramipril	ROR: 2.00; 95%CI: 1.09–3.68
Hepatic steatosis	Candesartan	ROR: 1.25; 95%CI: 0.66–2.39
Hepatic steatosis	Telmisartan	ROR: 0.82; 95%CI: 0.38–1.75
Hepatic steatosis	Valsartan	ROR: 0.79; 95%CI: 0.47–1.34
Hepatic steatosis	Hydrochlorothiazide	ROR: 0.72; 95%CI: 0.41–1.25
Hepatic steatosis	Indapamide	ROR: 1.25; 95%CI: 0.49–3.16
Blood triglycerides increased	Metoprolol	ROR: 0.41; 95%CI: 0.24–0.69
Blood triglycerides increased	Carvedilol	ROR: 0.98; 95%CI: 0.40–2.39
Blood triglycerides increased	Bisoprolol	ROR: 0.64; 95%CI: 0.35–1.17
Blood triglycerides increased	Nebivolol	ROR: 0.57; 95%CI: 0.22–1.50
Blood triglycerides increased	Enalapril	ROR: 1.97; 95%CI: 0.75–5.16
Blood triglycerides increased	Perindopril	ROR: 1.09; 95%CI: 0.45–2.68
Blood triglycerides increased	Lisinopril	ROR: 1.13; 95%CI: 0.54–2.37
Blood triglycerides increased	Ramipril	ROR: 1.17; 95%CI: 0.61–2.27
Blood triglycerides increased	Candesartan	ROR: 1.26; 95%CI: 0.54–2.92
Blood triglycerides increased	Valsartan	ROR: 0.36; 95%CI: 0.21–0.62
Blood triglycerides increased	Hydrochlorothiazide	ROR: 0.76; 95%CI: 0.36–1.58
High density lipoprotein decreased	Metoprolol	ROR: 0.87; 95%CI: 0.42–1.80
High density lipoprotein decreased	Bisoprolol	ROR: 0.83; 95%CI: 0.39–1.75
High density lipoprotein decreased	Perindopril	ROR: 0.98; 95%CI: 0.37–2.65
High density lipoprotein decreased	Lisinopril	ROR: 1.06; 95%CI: 0.46–2.45
High density lipoprotein decreased	Valsartan	ROR: 1.21; 95%CI: 0.48–3.06
High density lipoprotein decreased	Hydrochlorothiazide	ROR: 1.13; 95%CI: 0.42–3.05
Waist circumference increased	Bisoprolol	ROR: 0.36; 95%CI: 0.11–1.14
Metabolic syndrome	Metoprolol	ROR: 0.87; 95%CI: 0.31–2.44
Metabolic syndrome	Valsartan	ROR: 0.73; 95%CI: 0.24–2.17
Metabolic syndrome	Hydrochlorothiazide	ROR: 0.35; 95%CI: 0.14–0.92
Obesity	Metoprolol	ROR: 0.58; 95%CI: 0.35–0.96
Obesity	Carvedilol	ROR: 0.56; 95%CI: 0.29–1.08
Obesity	Bisoprolol	ROR: 2.17; 95%CI: 0.95–4.94
Obesity	Enalapril	ROR: 2.05; 95%CI: 0.85–4.93
Obesity	Perindopril	ROR: 0.82; 95%CI: 0.40–1.68
Obesity	Lisinopril	ROR: 0.28; 95%CI: 0.18–0.44
Obesity	Ramipril	ROR: 0.11; 95%CI: 0.07–0.16
Obesity	Candesartan	ROR: 0.05; 95%CI: 0.03–0.07
Obesity	Telmisartan	ROR: 0.80; 95%CI: 0.33–1.92
Obesity	Valsartan	ROR: 2.02; 95%CI: 0.84–4.86
Obesity	Hydrochlorothiazide	ROR: 1.05; 95%CI: 0.50–2.21

**Table 9:** PTs of interest related to metabolic disorders.

Pathophysiological Category	PT
Dysglycaemia and type 2 diabetes spectrum	Glucose tolerance impaired
Dysglycaemia and type 2 diabetes spectrum	Hyperglycaemia
Dysglycaemia and type 2 diabetes spectrum	Type 2 diabetes mellitus
Inflammatory Disorders & Inflammation biomarkers	C-reactive protein increased
Inflammatory Disorders & Inflammation biomarkers	Inflammatory marker increased
Inflammatory Disorders & Inflammation biomarkers	Inflammation
Metabolic syndrome & obesity-related disorders	Blood triglycerides increased
Metabolic syndrome & obesity-related disorders	High density lipoprotein decreased
Metabolic syndrome & obesity-related disorders	Waist circumference increased
Metabolic syndrome & obesity-related disorders	Metabolic syndrome
Metabolic syndrome & obesity-related disorders	Obesity
Metabolic syndrome & obesity-related disorders	Hepatic steatosis

## Data Availability

The original contributions presented in this study are included in the article. Further inquiries can be directed to the corresponding authors.

## Supplementary Materials

The following supporting information can be downloaded at: https://www.mdpi.com/article/10.3390/ph19081177/s1, Table S1: Number of reports for dysglycaemia and T2DM spectrum; Table S2: Inflammatory disorders and inflammation biomarkers; Table S3: Metabolic syndrome and obesity-related disorders.

## Abbreviations

The following abbreviations are used in this manuscript:

ACE	Angiotensin-converting enzyme
ADR	Adverse drug reactions
BB	Beta-blockers
BRA	Angiotensin II receptor blockers
CCB	Calcium channel blockers
CI	Confidence interval
EEA	European Economic Area
EV	EudraVigilance
HP	Healthcare professionals
ICSR	Individual Case Safety Reports
MedDRA	Medical Dictionary for Regulatory Activities
RAAS	Renin–angiotensin–aldosterone system
ROR	Reporting odds ratio
SmPC	Summary of the Product Characteristics
T2DM	Type 2 diabetes mellitus
CVD	Cardiovascular disease
MS	Metabolic syndrome
CRP	C-reactive protein
DHP	Dihydropyridine
