# Supplementary material for: Metabolic and Inflammatory Adverse Drug Reactions Associated with Amlodipine: A Descriptive and Disproportionality Analysis of EudraVigilance Reports
Source: Pharmaceuticals (Basel). 2026 Jul 27;19(8):1177. doi: 10.3390/ph19081177 (PMC13516587; doi:10.3390/ph19081177)
Supplement: Supplementary file 1 [file pharmaceuticals-19-01177-s001.zip › pharmaceuticals-4443230-supplementary.pdf]

Supplementary Material

Table S1 – Number of reports for dysglycaemia and T2DM spectrum

PT	Amlodipine			Comparator drug			
	**Total ADRs**	**ADRs of interest**	**Other ADRs**	**Drug**	**Total ADRs**	**ADRs of interest**	**Other ADRs**
glucose tolerance impaired	32560	12	32548	Metoprolol	18858	10	18848
glucose tolerance impaired	32560	12	32548	Carvedilol	7946	7	7939
glucose tolerance impaired	32560	12	32548	Enalapril	13357	5	13352
glucose tolerance impaired	32560	12	32548	Lisinopril	15384	8	15376
glucose tolerance impaired	32560	12	32548	Valsartan	13165	11	13154
glucose tolerance impaired	32560	12	32548	Hydrochlorothiazide	10254	7	10247
hyperglycaemia	32560	136	32424	Nifedipine	8798	30	8768
hyperglycaemia	32560	136	32424	Felodipidine	2219	6	2213
hyperglycaemia	32560	136	32424	Metoprolol	18858	48	18810
hyperglycaemia	32560	136	32424	Carvedilol	7946	46	7900
hyperglycaemia	32560	136	32424	Bisoprolol	16482	27	16455
hyperglycaemia	32560	136	32424	Enalapril	13357	22	13335
hyperglycaemia	32560	136	32424	Perindopril	8903	7	8896
hyperglycaemia	32560	136	32424	Lisinopril	15384	37	15347
hyperglycaemia	32560	136	32424	Ramipril	22255	33	22222
hyperglycaemia	32560	136	32424	Candesartan	11939	13	11926
hyperglycaemia	32560	136	32424	Telmisartan	5213	16	5197
hyperglycaemia	32560	136	32424	Valsartan	13165	39	13126
hyperglycaemia	32560	136	32424	Hydrochlorothiazide	10254	43	10211
hyperglycaemia	32560	136	32424	Indapamide	4952	14	4938
type 2 diabetes mellitus	32560	41	32519	Nifedipine	8798	5	8793
type 2 diabetes mellitus	32560	41	32519	Metoprolol	18858	51	18807
type 2 diabetes mellitus	32560	41	32519	Carvedilol	7946	17	7929

type 2 diabetes mellitus	32560	41	32519	Bisoprolol	16482	19	16463
type 2 diabetes mellitus	32560	41	32519	Nebivolol	3886	5	3881
type 2 diabetes mellitus	32560	41	32519	Enalapril	13357	8	13349
type 2 diabetes mellitus	32560	41	32519	Perindopril	8903	12	8891
type 2 diabetes mellitus	32560	41	32519	Lisinopril	15384	93	15291
type 2 diabetes mellitus	32560	41	32519	Ramipril	22255	276	21979
type 2 diabetes mellitus	32560	41	32519	Candesartan	11939	295	11644
type 2 diabetes mellitus	32560	41	32519	Telmisartan	5213	8	5205
type 2 diabetes mellitus	32560	41	32519	Valsartan	13165	45	13120
type 2 diabetes mellitus	32560	41	32519	Hydrochlorothiazide	10254	10	10244
type 2 diabetes mellitus	32560	41	32519	Indapamide	4952	13	4939

Table S2 – Inflammatory disorders and inflammation biomarkers

	**Amlodipine**			**Comparator drug**			
**PT**	**Total ADRs**	**ADRs of interest**	**Other ADRs**	**Drug**	**Total ADRs**	**ADRs of interest**	**Other ADRs**
C-reactive protein increased	32,560	80	32,480	Nifedipine	8,798	16	8,782
C-reactive protein increased	32,560	80	32,480	Metoprolol	18,858	22	18,836
C-reactive protein increased	32,560	80	32,480	Carvedilol	7,946	28	7,918
C-reactive protein increased	32,560	80	32,480	Bisoprolol	16,482	52	16,430
C-reactive protein increased	32,560	80	32,480	Enalapril	13,357	25	13,332
C-reactive protein increased	32,560	80	32,480	Perindopril	8,903	19	8,884
C-reactive protein increased	32,560	80	32,480	Lisinopril	15,384	38	15,346
C-reactive protein increased	32,560	80	32,480	Ramipril	22,255	194	22,061
C-reactive protein increased	32,560	80	32,480	Candesartan	11,939	256	11,683
C-reactive protein increased	32,560	80	32,480	Telmisartan	5,213	20	5,193
C-reactive protein increased	32,560	80	32,480	Valsartan	13,165	115	13,050
C-reactive protein increased	32,560	80	32,480	Hydrochlorothiazide	10,254	21	10,233

C-reactive protein increased	32,560	80	32,480	Indapamide	4,952	9	4,943
Inflammation	32,560	50	32,510	Nifedipine	8,798	10	8,788
Inflammation	32,560	50	32,510	Felodipine	2,219	6	2,213
Inflammation	32,560	50	32,510	Metoprolol	18,858	42	18,816
Inflammation	32,560	50	32,510	Carvedilol	7,946	7	7,939
Inflammation	32,560	50	32,510	Bisoprolol	16,482	24	16,458
Inflammation	32,560	50	32,510	Nebivolol	3,886	5	3,881
Inflammation	32,560	50	32,510	Enalapril	13,357	8	13,349
Inflammation	32,560	50	32,510	Perindopril	8,903	26	8,877
Inflammation	32,560	50	32,510	Lisinopril	15,384	33	15,351
Inflammation	32,560	50	32,510	Ramipril	22,255	266	21,989
Inflammation	32,560	50	32,510	Candesartan	11,939	279	11,660
Inflammation	32,560	50	32,510	Valsartan	13,165	47	13,118
Inflammation	32,560	50	32,510	Hydrochlorothiazide	10,254	16	10,238
Inflammatory marker increased	32,560	13	32,547	Bisoprolol	16,482	11	16,471
Inflammatory marker increased	32,560	13	32,547	Ramipril	22,255	5	22,250

Table S3 - Metabolic syndrome and obesity-related disorders

	**Amlodipine**			**Comparator drug**			
**PT**	**Total ADRs**	**ADRs of interest**	**Other ADRs**	**Drug**	**Total ADRs**	**ADRs of interest**	**Other ADRs**
hepatic steatosis	32,560	41	32,519	Nifedipine	8,798	5	8,793
hepatic steatosis	32,560	41	32,519	Metoprolol	18,858	24	18,834
hepatic steatosis	32,560	41	32,519	Carvedilol	7,946	8	7,938
hepatic steatosis	32,560	41	32,519	Bisoprolol	16,482	12	16,470
hepatic steatosis	32,560	41	32,519	Enalapril	13,357	13	13,344
hepatic steatosis	32,560	41	32,519	Perindopril	8,903	8	8,895
hepatic steatosis	32,560	41	32,519	Lisinopril	15,384	23	15,361
hepatic steatosis	32,560	41	32,519	Ramipril	22,255	14	22,241

hepatic steatosis	32,560	41	32,519	Candesartan	11,939	12	11,927
hepatic steatosis	32,560	41	32,519	Telmisartan	5,213	8	5,205
hepatic steatosis	32,560	41	32,519	Valsartan	13,165	21	13,144
hepatic steatosis	32,560	41	32,519	Hydrochlorothiazid e	10,254	18	10,236
hepatic steatosis	32,560	41	32,519	Indapamide	4,952	5	4,947
blood triglycerides increased	32,560	24	32,536	Metoprolol	18,858	34	18,824
blood triglycerides increased	32,560	24	32,536	Carvedilol	7,946	6	7,940
blood triglycerides increased	32,560	24	32,536	Bisoprolol	16,482	19	16,463
blood triglycerides increased	32,560	24	32,536	Nebivolol	3,886	5	3,881
blood triglycerides increased	32,560	24	32,536	Enalapril	13,357	5	13,352
blood triglycerides increased	32,560	24	32,536	Perindopril	8,903	6	8,897
blood triglycerides increased	32,560	24	32,536	Lisinopril	15,384	10	15,374
blood triglycerides increased	32,560	24	32,536	Ramipril	22,255	14	22,241
blood triglycerides increased	32,560	24	32,536	Candesartan	11,939	7	11,932
blood triglycerides increased	32,560	24	32,536	Valsartan	13,165	27	13,138
blood triglycerides increased	32,560	24	32,536	Hydrochlorothiazid e	10,254	10	10,244
High density lipoprotein decreased	32,560	18	32,542	Metoprolol	18,858	12	18,846
High density lipoprotein decreased	32,560	18	32,542	Bisoprolol	16,482	11	16,471
High density lipoprotein decreased	32,560	18	32,542	Perindopril	8,903	5	8,898
High density lipoprotein decreased	32,560	18	32,542	Lisinopril	15,384	8	15,376
High density lipoprotein decreased	32,560	18	32,542	Valsartan	13,165	6	13,159
High density lipoprotein decreased	32,560	18	32,542	Hydrochlorothiazid e	10,254	5	10,249
waist circumference increased	32,560	5	32,555	Bisoprolol	16,482	7	16,475

metabolic syndrome	32,560	`	32,551	Metoprolol	18,858	6	18,852
metabolic syndrome	32,560	9	32,551	Valsartan	13,165	5	13,160
metabolic syndrome	32,560	9	32,551	Hydrochlorothiazide	10,254	8	10,246
obesity	32,560	30	32,530	Metoprolol	18,858	30	18,828
obesity	32,560	30	32,530	Carvedilol	7,946	13	7,933
obesity	32,560	30	32,530	Bisoprolol	16,482	7	16,475
obesity	32,560	30	32,530	Enalapril	13,357	6	13,351
obesity	32,560	30	32,530	Perindopril	8,903	10	8,893
obesity	32,560	30	32,530	Lisinopril	15,384	50	15,334
obesity	32,560	30	32,530	Ramipril	22,255	186	22,069
obesity	32,560	30	32,530	Candesartan	11,939	222	11,717
obesity	32,560	30	32,530	Telmisartan	5,213	6	5,207
obesity	32,560	30	32,530	Valsartan	13,165	6	13,159
obesity	32,560	30	32,530	Hydrochlorothiazide	10,254	9	10,245
